# Supplementary material for: Optical imaging of the intrinsic adsorption kinetics in single zeolite nanoparticles
Source: Nat Commun. 2026 Mar 11;17:3811. doi: 10.1038/s41467-026-70625-7 (PMC13111643; doi:10.1038/s41467-026-70625-7)
Supplement: Supplementary file 1 — Supplementary Information [file 41467_2026_70625_MOESM1_ESM.pdf]

Supplementary Information

Optical imaging of the intrinsic adsorption kinetics in single zeolite nanoparticles

Xuannuo Yi[1], Haoran Han[1], Aosheng Chang[1], Ziyuan Liu[2], Qingxue Hui[3], Chongqin Zhu[2], Zhaoqiang Zhang[3], Shasha Liu[1,4*], Wei Wang[1*]

[1] State Key Laboratory of Analytical Chemistry for Life Science, Chemistry and Biomedicine Innovation Center (ChemBIC), School of Chemistry and Chemical Engineering, Nanjing University, Nanjing 210023, China

[2] College of Chemistry, Key Laboratory of Theoretical & Computational Photochemistry of Ministry of Education, Beijing Normal University, Beijing 100190, China

[3] State Key Laboratory of Coordination Chemistry, School of Chemistry and Chemical Engineering, Nanjing University, Nanjing 210023, China

[4] Shenzhen Research Institute of Nanjing University, Shenzhen 518000, China

[*]Correspondence should be addressed to S.L. (ssliu@nju.edu.cn) and W.W. (wei.wang@nju.edu.cn)

Table of Contents

Supplementary Figures

1. Comprehensive characterizations of the ZSM-5 particles
2. Detailed schematic diagram of the experimental set-up
3. Correlated optical dark-field microscopy (DFM) images with scanning electron microscopy (SEM) images of ZSM-5 particles
4. Effect of gas flow rate on k_a and k_d of single ZSM-5 nanoparticles
5. Bulk *in situ* IR measurement of ZSM-5 before and adsorption of C_3H_6
6. Optical response curves of a single ZSM-5 nanoparticle during an adsorption-desorption cycle of CO_2 or O_2
7. Optical response curves of single Si nanoparticles during an adsorption-desorption cycle of C_3H_6
8. Extraction and quantitative analysis of the optical response of single ZSM-5 nanoparticles during the adsorption-desorption cycle
9. Determining the elementary adsorption kinetics using the pseudo-first-order kinetics model
10. Dependence of $k_{obs,a}$ and $k_{obs,d}$ on the C_3H_6 concentration
11. The dependence of $k_{obs,a}$ on the particle size of the ZSM-5 nanoparticles used in this work
12. Comparison of the size dependence of $k_{obs,a}$ among ZSM-5 particles with different Si/Al ratio
13. The K_A values of three olefins extracted from each ZSM-5 nanoparticle
14. The dependence of k_a on the particle size of single ZSM-5 particles of three olefins
15. The relationship between k_d, K_A and protonation energies
16. Determination of the adsorption and desorption activation energies of C_2H_4
17. Determination of the adsorption and desorption activation energies of n-C_4H_8
18. Relationship between temperatures and alkene adsorption amounts
19. Cyclic adsorption-desorption experiments of three olefin molecules
20. Bulk characterizations of the HY and SSZ-13 particles

21. Comparison of the adsorption kinetics and thermodynamics of light alcohols

Supplementary Tables

1. Bulk characterizations of the ZSM-5 particles with different Si/Al ratios

2. The structural information of zeolites used in adsorption-desorption experiments

References

Supplementary Figures

1. Comprehensive characterizations of the ZSM-5 particles

The transmission electron microscopy (TEM) images with energy-dispersive X-ray (EDX) mapping shown in Supplementary Fig. 1a clearly demonstrate the coffin-like crystal morphology of the ZSM-5 particles used in this work. Supplementary Fig. 1b exhibits the X-ray Diffraction (XRD) pattern of the ZSM-5 particles with distinct diffraction reflections at 2θ = 7.9, 8.8, 23.2, 23.9, and 24.4°, which is well aligned to a typical MFI zeolite structure. The acidity of ZSM-5 zeolites was assessed using NH_3-temperature programmed desorption (TPD). Two peaks at around 199°C and 433°C were clearly observed in the spectrum as demonstrated in Supplementary Fig. 1c, which correspond to the desorption of NH_3 from weak and strong acid sites on ZSM-5, respectively. N_2 adsorption/desorption isotherm was conducted to determine the textural properties of ZSM-5, revealing a surface area of 352 m²/g. The chemical composition was determined by an inductively coupled plasma optical emission spectrum (ICP-OES), and the determined Si/Al ratio (52.1) was in good agreement with the values provided by the supplier (50). Clarify that the ZSM-50 with a Si/Al ratio of 50 is the main material used in this work.

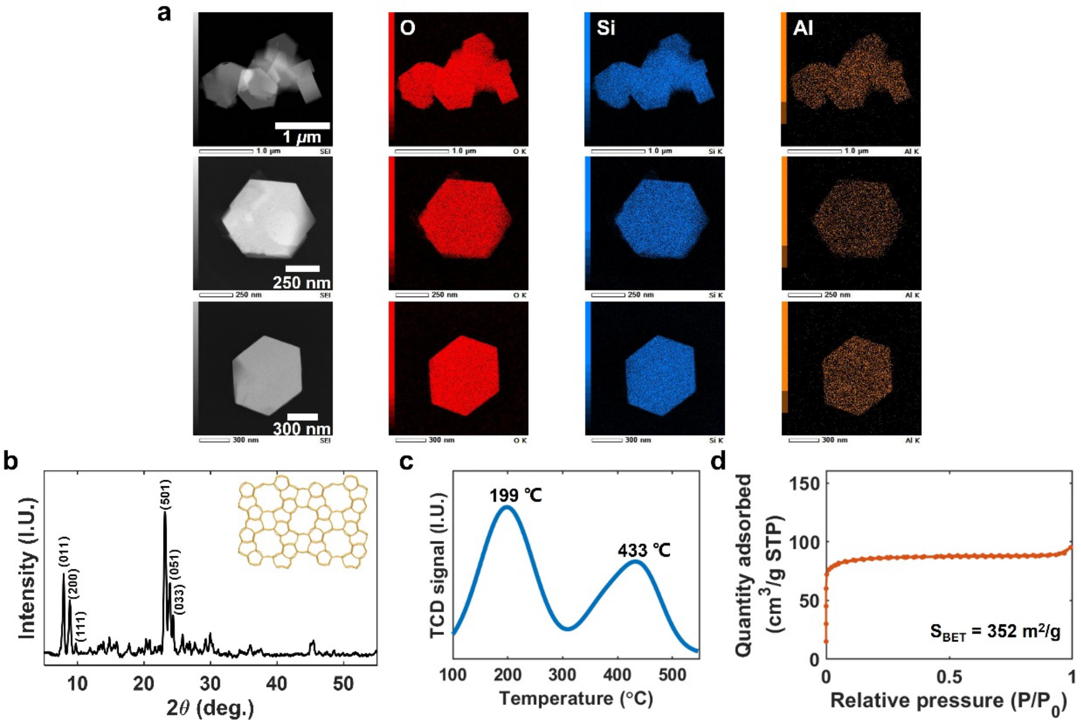

Supplementary Fig. 1. Characterization results of the ZSM-5 particles used in this work. **(a)** TEM-EDX images. **(b)** XRD pattern. **(c)** NH_3-TPD spectrum. **(d)** N_2 adsorption/desorption isotherm.

2. Detailed schematic diagram of the experimental set-up

The detailed schematic diagram of the experimental setup is shown in Supplementary Fig. 2, which consists of three main components—the gas-flow system for controlling the gas atmosphere around ZSM-5 nanoparticles and thus triggering the adsorption and desorption process, the optical imaging system for monitoring the whole adsorption process of ZSM-5 on a single nanoparticle level, and the micro-area-heating module for temperature control as well as the data record system. More details about the introduction of each component could be found in our previous work.[1]

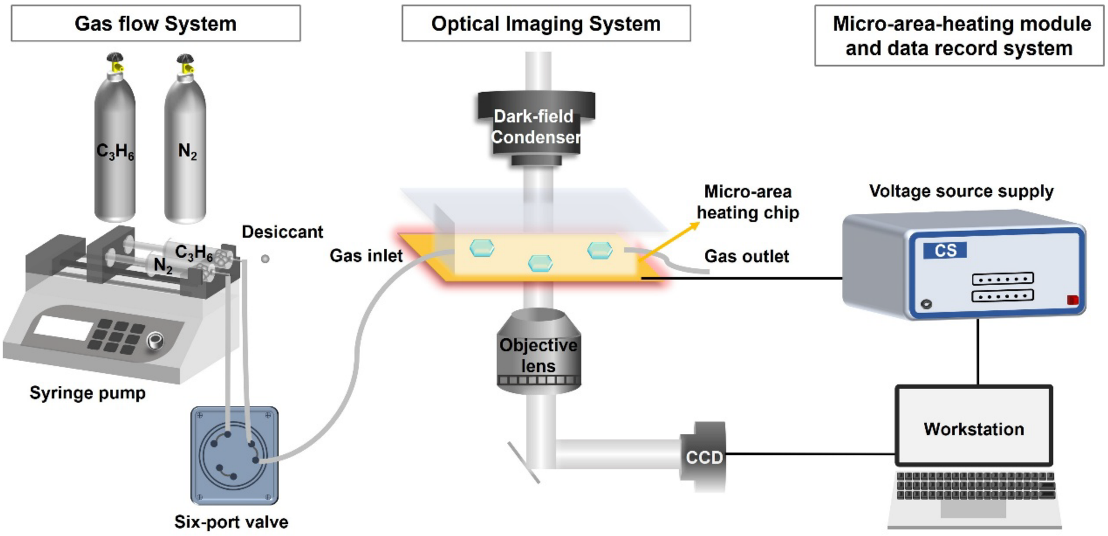

Supplementary Fig. 2. Schematic diagram of the experimental set-up.

3. Correlated optical dark-field microscopy (DFM) images with scanning electron microscopy (SEM) images of ZSM-5 particles

After taking the DFM image of the ZSM-5 nanoparticles, we further imaged the same area in SEM with the help of a pre-made marker. The comparison of the DFM images and SEM images of the very same ZSM-5 particles not only confirmed that each bright spot in the DFM image represented a single ZSM-5 particle, but also suggested that the ZSM-5 with a bigger size exhibited a higher optical intensity in the DFM as described by Mie theory.[2,3]

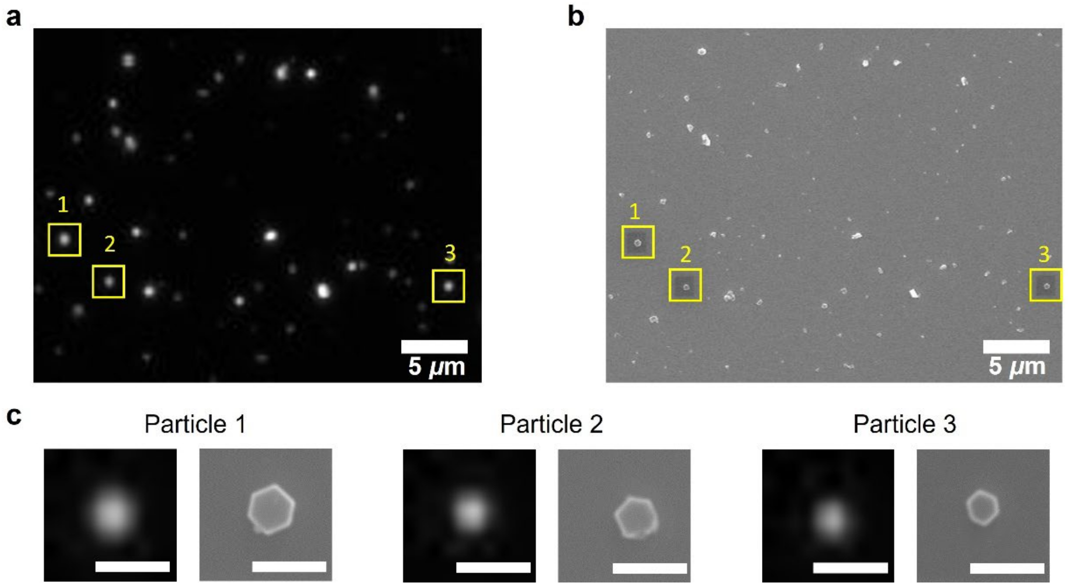

Supplementary Fig. 3. DFM image **(a)** and correlative SEM image **(b)** of tens of ZSM-5 particles in the same field view. Scale bar: 5 μm. **(c)** The result of three representative particles. Scale bar: 1 μm.

4. Effect of gas flow rate on k_a and k_d of single ZSM-5 nanoparticles

To minimize the impact of mass transfer limitations on the determination of the intrinsic adsorption kinetics of single ZSM-5 nanoparticles, adsorption-desorption cycles of the same ZSM-5 particle were obtained under different gas flow rates to establish an optimal experimental condition (Supplementary Fig. 4a). It was found that the adsorption rate constant (k_a) initially increased with the flow rates within the range of 0.5-3 mL/min, but leveled off as the flow rates continuously increased, suggesting a fast enough mass transfer process to have negligible influence on the experimental results (Supplementary Fig. 4b). By contrast, no clear dependence of the desorption rate constant was observed on the flow rate of gases (Supplementary Fig. 4c). To further balance the influence of gas flow on the mass transfer in the adsorption process and the stability of the imaging system, 3 mL/min was chosen as the final gas flow rate for all other experiments shown in this work.

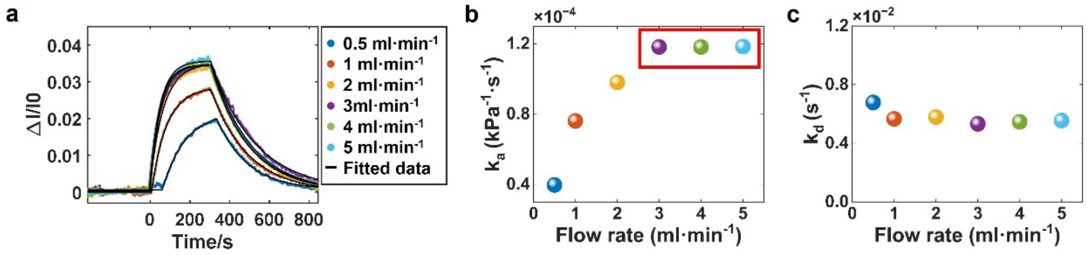

Supplementary Fig. 4. (a) The adsorption-desorption curves of C_3H_6 on a single ZSM-5 nanoparticle under different flow rates of gas. **(b-c)** The scatter diagrams of the experimentally determined k_a and k_d as a function of corresponding gas flow rates.

5. Bulk *in situ* IR measurement of ZSM-5 before and adsorption of C_3H_6

To investigate the interaction between ZSM-5 and C_3H_6, *in situ* IR spectroscopy was conducted on ZSM-5 powder. As shown in Supplementary Fig. 5, after 30 min of C_3H_6 adsorption at 100 °C the differential IR spectrum of bulk ZSM-5—obtained by subtracting the pre-adsorption spectrum from the post-adsorption spectrum—exhibits a pronounced negative band at 3610 cm^{-1}, corresponding to the consumption of Brønsted acidic OH groups. Simultaneously, the occurrence of bands at 2955, 2930, and 2860 cm^{-1} ($\nu(CH_3)/\nu(CH_2)$) and 1470 cm^{-1} ($\delta(CH)$) indicate the formation of methyl/methylene species. These results demonstrate that C3H6 interacts primarily with Brønsted acid sites.[4,5]

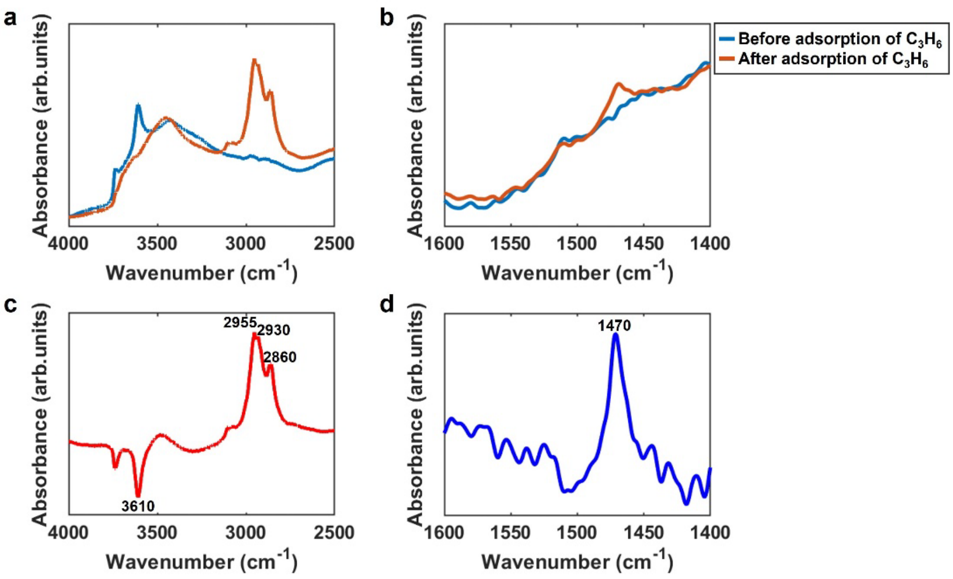

Supplementary Fig. 5. (a-b) The original *in-situ* IR adsorbance spectra before and after adsorption of C_3H_6 at 100 °C. **(c-d)** The differential IR spectrum of bulk ZSM-5—obtained by subtracting the pre-adsorption spectrum from the post-adsorption spectrum.

6. Optical response curves of a single ZSM-5 nanoparticle during an adsorption-desorption cycle of CO_2 or O_2

To ensure that the increased scattering intensity of single ZSM-5 nanoparticles originated from the adsorption and desorption of C_3H_6, control experiments were conducted on the very same individual ZSM-5 particle by substituting C_3H_6 with CO_2 or O_2. No significant change in the optical intensity of the ZSM-5 particle was observed for either CO_2 or O_2. Note that the small change in the optical intensity possibly resulted from the slight vibrations in the imaging system at the moment of gas switching.

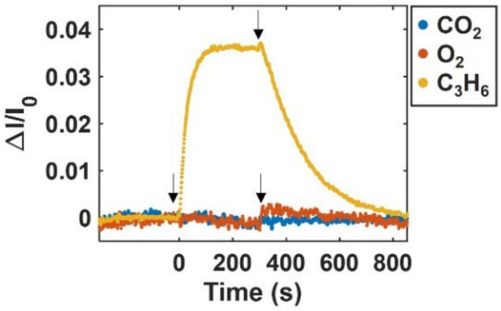

Supplementary Fig. 6. Optical curves of the very same ZSM-5 nanoparticle during the adsorption-desorption cycle of C_3H_6 (yellow), CO_2 (blue), and O_2 (orange), respectively.

7. Optical response curves of single Si nanoparticles during an adsorption-desorption cycle of C_3H_6

The control experiment was also performed on non-acidic Si nanoparticles. The micro-Raman spectrum confirms the existence of a single Si nanoparticle, as displayed in Supplementary Fig. 7a. No signals were observed in their adsorption and desorption processes of C_3H_6, which is reasonable due to the absence of acidic sites in the Si particles. This result also suggested that physical adsorption may not contribute to the optical intensity change in this study.

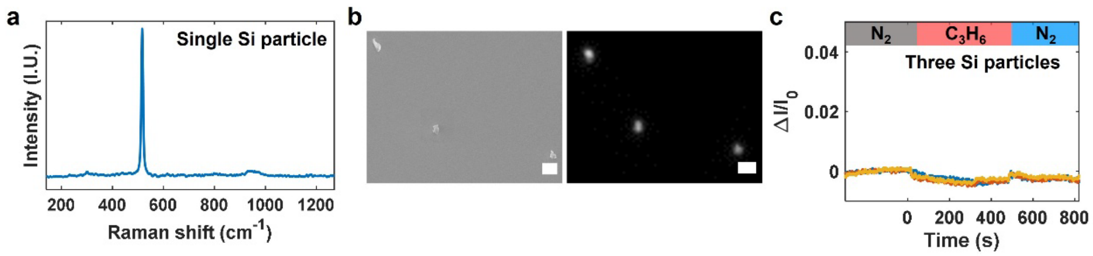

Supplementary Fig. 7. (a) Raman spectrum of a single Si nanoparticle. The wavelength of the laser is 633 nm, and the acquisition time of the spectrum is 1 s. **(b)** DFM image and correlative SEM image of single Si nanoparticles. Scale bar: 1 μm. **(c)** Optical curves of three representative Si nanoparticles during an adsorption-desorption cycle of C_3H_6.

8. Extraction and quantitative analysis of the optical response of single ZSM-5 nanoparticles during the adsorption-desorption cycle

Supplementary Fig. 8a shows the original optical curves of a single ZSM-5 particle during an adsorption-desorption cycle, which was extracted from the time-lapsed DFM images. Specifically, a region of interest (ROI, 13*13 pixels) that covers the entire particle was selected, and the average value of all pixels in the ROI represented the optical intensity of the ZSM-5 at each frame (time point). The change in optical intensity (ΔI) was further normalized by the initial intensity (I_0) of particles to remove the size effect. Therefore, $\Delta I/I_0$ represents the relative adsorption capacity of C_3H_6 in each ZSM-5 nanoparticle. All the data analysis was automatically processed by MATLAB.

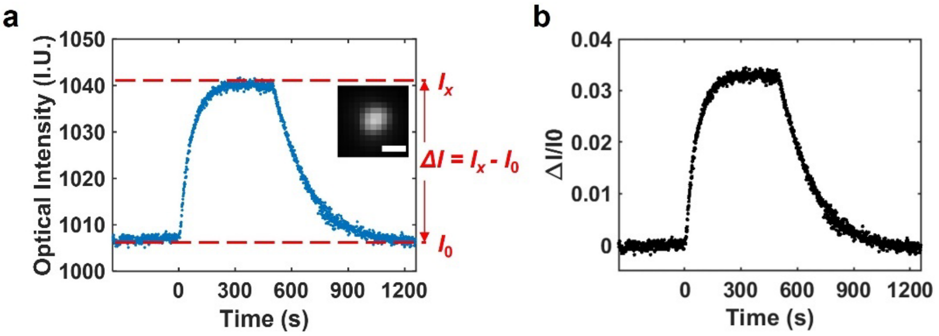

Supplementary Fig. 8. Corresponding original **(a)** and normalized **(b)** optical curves of a single ZSM-5 nanoparticle during the adsorption-desorption of C_3H_6. The inset image in **(a)** demonstrates the ROI used for the quantitative analysis and the scale bar is 1 μm.

9. Determining the elementary adsorption kinetics using the pseudo-first-order kinetics model

To determine the adsorption kinetics mechanism, we further plotted ln ($I_e - I_t$) as a function of time, and a linear relationship was found in both the adsorption and desorption processes. The result suggested the interaction between the acid sites of ZSM-5 and C_3H_6 followed pseudo-first-order kinetics.

The equation can be written as

$$C_3H_6 + \text{Zeolite-O-H} \underset{k_d}{\overset{k_a}{\rightleftarrows}} \text{Zeolite-O-}C_3H_7 \qquad (1)$$

During the adsorption phase, the complex [Zeolite-O-C_3H_7] increases as a function of time according to

$$\frac{d[\text{Zeolite-O-}C_3H_7]}{dt} = k_a \cdot [C_3H_6] \cdot [\text{Zeolite-O-H}] - k_d \cdot [\text{Zeolite-O-}C_3H_7] \qquad (2)$$

Where k_a ($kPa^{-1} \cdot s^{-1}$) and k_d (s^{-1}) are the elementary adsorption rate constant and desorption rate constant. Define R_t as the value of signal change resulting from the adsorption of C_3H_6 on the zeolite at time t, R_{max} is the maximum response value at adsorption saturation. Then, the equation can be transformed into

$$\frac{d[R_t]}{dt} = k_a \cdot [C_3H_6] \cdot (R_{max} - R_t) - k_d \cdot [R_t] \qquad (3)$$

$$R_t = \frac{R_{max} \cdot [C_3H_6]}{K_D + [C_3H_6]} [1 - e^{(k_a \cdot [C_3H_6] + k_d) \cdot t)}] \qquad (4)$$

where the equilibrium constant K_A is calculated from the two kinetic rate constants through the relation $K_A = \frac{k_a}{k_d}$. According to the pseudo-first-order chemical adsorption model, the relationship between $k_{obs,a}$ and k_a, k_d is derived

$$k_{obs,a} = k_a \cdot P + k_d \qquad (5)$$

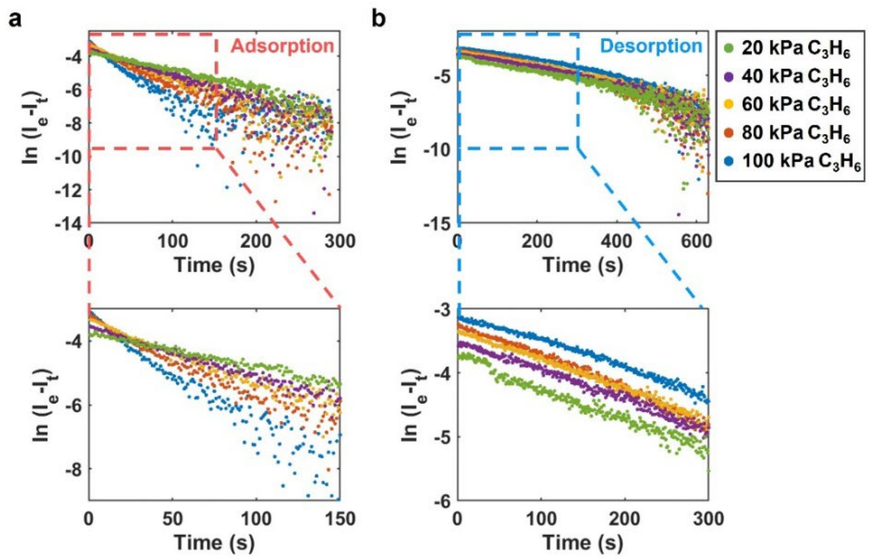

Supplementary Fig. 9. The plot of ln (I_e−I_t) versus t for the adsorption and desorption processes.

Although the pseudo-first-order reversible adsorption model provides a good fit to the experimental data the inherent limitations should be acknowledged. The model assumes that all Brønsted acidic sites on ZSM-5 nanoparticles are homogeneous in strength, which is an ideal simplification. The local coordination environment, framework topology, and possible defects can lead to a distribution of acid site strengths even within the same nanoparticle. However, such fine-scale heterogeneity cannot be resolved with the current temporal and spatial resolution of our single-particle measurement technique. Despite this limitation, the pseudo-first-order reversible model successfully captures the dominant kinetic behavior and allows for reliable comparison of adsorption kinetics across different adsorbates and confinement environments.

10. Dependence of $k_{obs,a}$ and $k_{obs,d}$ on the C_3H_6 concentration

To further validate the previously proposed adsorption and desorption mechanisms, we explored how observed adsorption and desorption kinetics changed as the concentration of C_3H_6, which was achieved by changing the partial pressure of C_3H_6 in the gas flow. The statistical result of 79 ZSM-5 particles in Supplementary Fig. 10, clearly suggested that the k_{obs} for adsorption linear increase as the partial pressures of C_3H_6, and no obvious change was found in the desorption. These results further supported the pseudo-first-order adsorption kinetics mechanism. Moreover, this discrepancy in the dependence of k_{obs} on the partial pressure of C_3H_6 during adsorption and desorption, further ruled out diffusion as the primary contributor to the observed optical response.

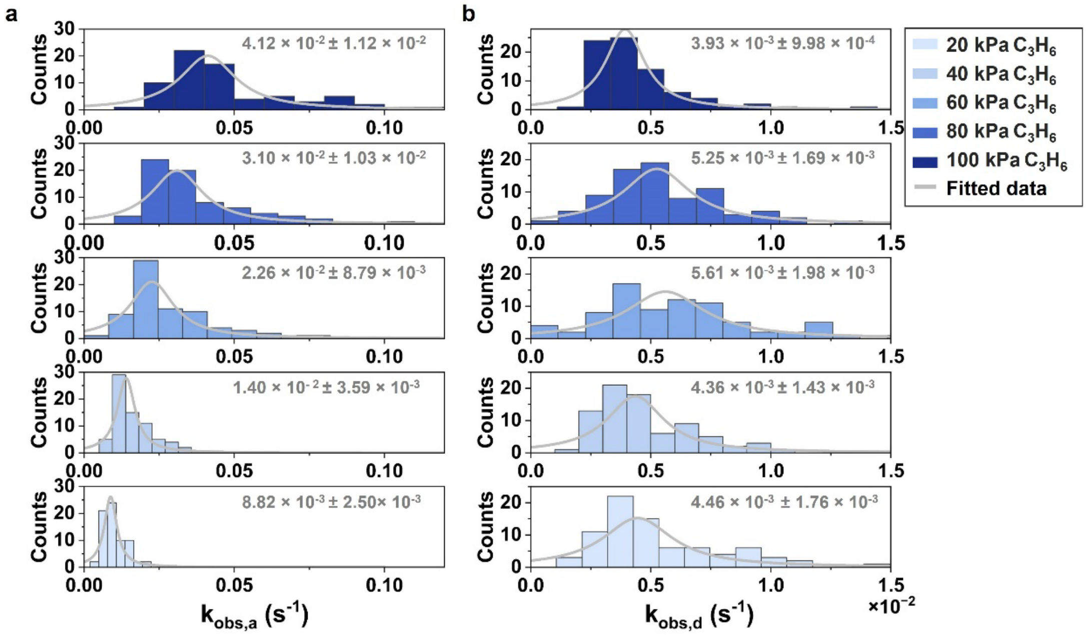

Supplementary Fig. 10. (a-b) Statistical histogram of $k_{obs,a}$ and $k_{obs,d}$ under different partial pressures of C_3H_6.

11. The dependence of $k_{obs,a}$ on the particle size of the ZSM-5 nanoparticles used in this work

As mentioned previously, the ZSM-5 nanoparticles with a bigger size show a higher original DFM optical intensity, therefore, the DFM intensity can be used to describe the size of ZSM-5 nanoparticles. When plotting $k_{obs,a}$ and $k_{obs,d}$ versus I_0 of 50 particles obtained in one experiment, it was found that there was no clear dependence between rate constants and particle size (Supplementary Fig. 11a and 11b). The optical adsorption-desorption curves of two representative particles are shown in Supplementary Fig. 11c and 11d, which differed in size by nearly a factor of two but exhibited very close rate constants, serving as the direct evidence that the adsorption and desorption kinetics of C_3H_6 on ZSM-5 with a Si/Al ratio of 50 are not dominated by diffusion.

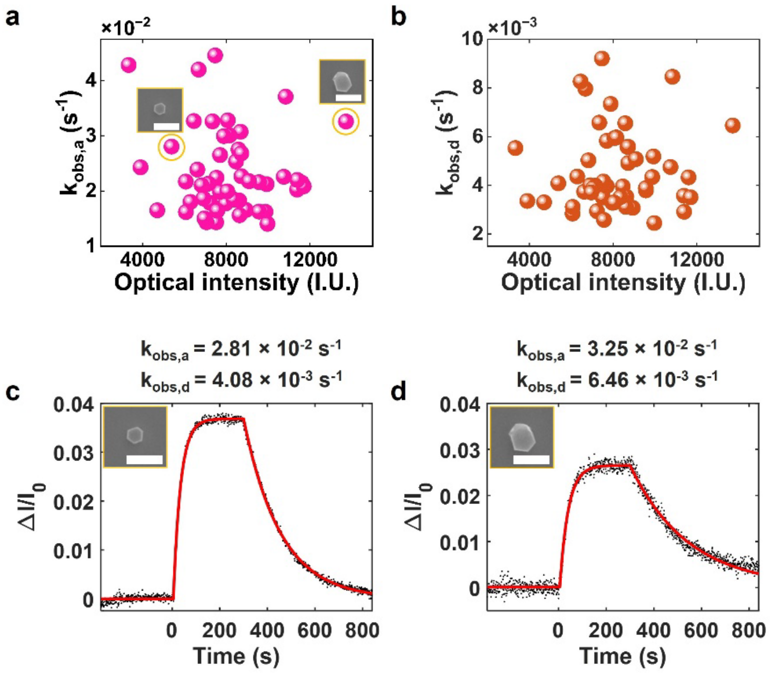

Supplementary Fig. 11. (a-b) Scatter diagrams of $k_{obs,a}$ and $k_{obs,d}$ versus I_0 of single ZSM-5 nanoparticles. **(c-d)** The adsorption-desorption curves of two ZSM-5 nanoparticles labeled in **(a)**. Scale bar: 1 μm.

12. Comparison of the size dependence of $k_{obs,a}$ among ZSM-5 particles with different Si/Al ratio

To further explore the diffusion effect in a single nanoparticle, the size dependence of adsorption-desorption kinetics of C_3H_6 on particles with the same MFI framework, similar particle size range, but different acidity, by modulating the Si/Al ratio in particles. The dependence between $k_{obs,a}$ and particle size gradually appeared as the Si/Al ratio, i.e., acidity decreased. The result suggests that diffusion does not play a dominant role in the adsorption kinetics on particles with strong acidity. It is worth noting that the ZSM-5 with a Si/Al ratio of 50, which exhibits the strongest acidity among the three samples, is the particle investigated in this study. In contrast, the adsorption of C_3H_6 on Silicalite-1 is a diffusion-limited process, as evidenced by the clear particle-size dependence of the adsorption rates—larger Silicalite-1 particles require longer times to reach equilibrium.

Prior to the measurement of adsorption kinetics, the zeolites with same MFI framework, similar particle size range, but different Si/Al ratio was characterized. The XRD patterns of these particles exhibited the similar peak features, supporting that the same MFI framework. Note that the result of ZSM-5 with a Si/Al ratio of 50 (ZSM-5-50) was listed in Supplementary Fig. 1.

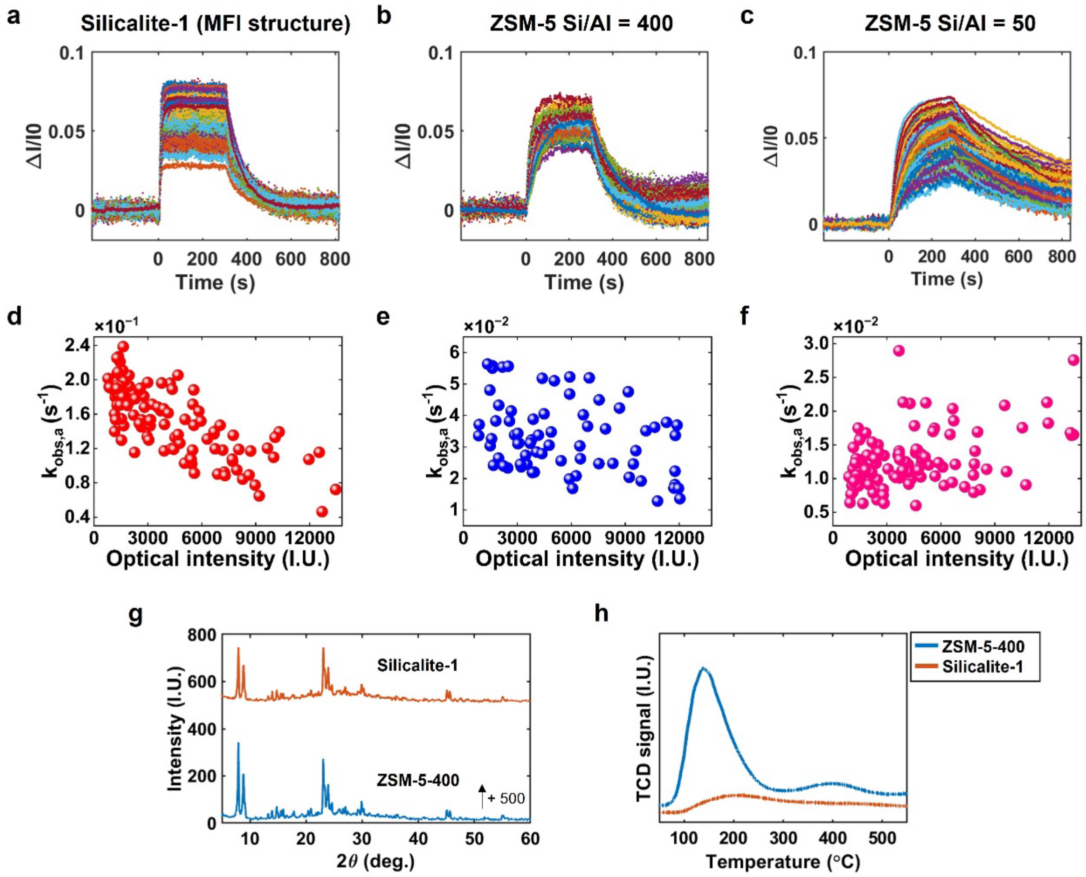

Supplementary Fig. 12. The adsorption-desorption curves of silicalite-1 **(a)** ZSM-5 with a Si/Al ratio of 400 **(b)** and 50 **(c)** at 70 °C. Scatter diagrams of $k_{obs,a}$ versus I_0 of silicalite-1 **(d)**, ZSM-5 with a Si/Al ratio of 400 **(e)**, and 50 **(f)**. **(g-h)** XRD patterns and NH_3-TPD spectra of the ZSM-5 with Si/Al ratio of 400 and silicalite-1

particles used in this work.

13. The K_A values of three olefins extracted from each ZSM-5 nanoparticle

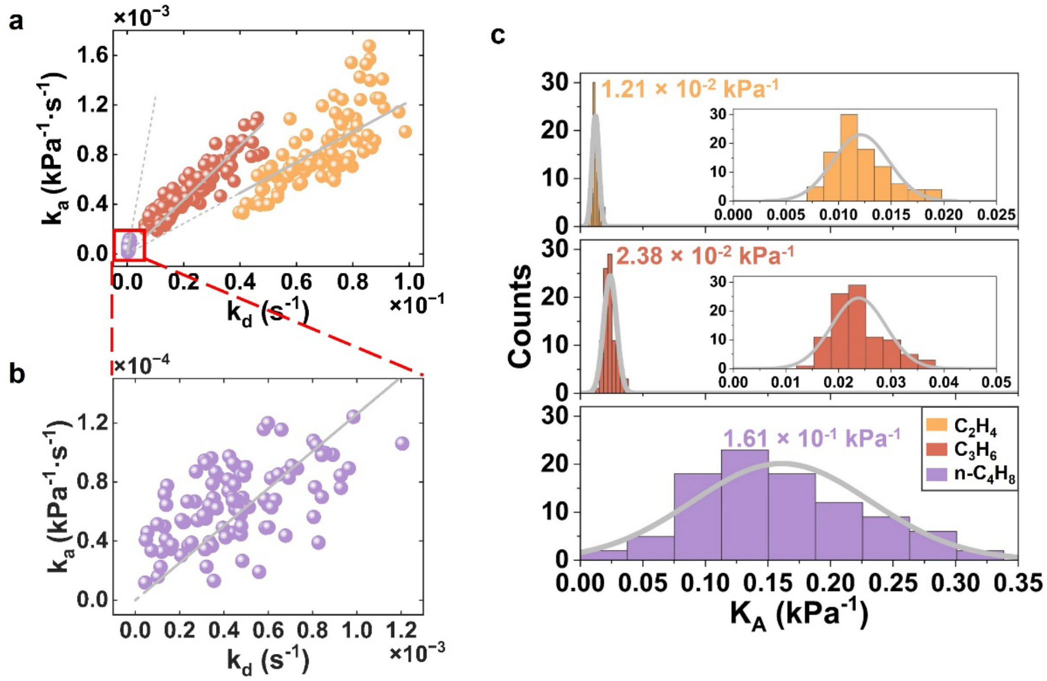

Supplementary Fig. 13. (a-b) The statistical relationship between the rate constants for the elementary adsorption and desorption steps of each olefin molecule, based on the response of 103 ZSM-5 nanoparticles. Each point represents the result from one particle. **(c)** Statistical histograms of K_A values of three olefins of each particle.

14. The dependence of k_a on the particle size of single ZSM-5 particles of three olefins

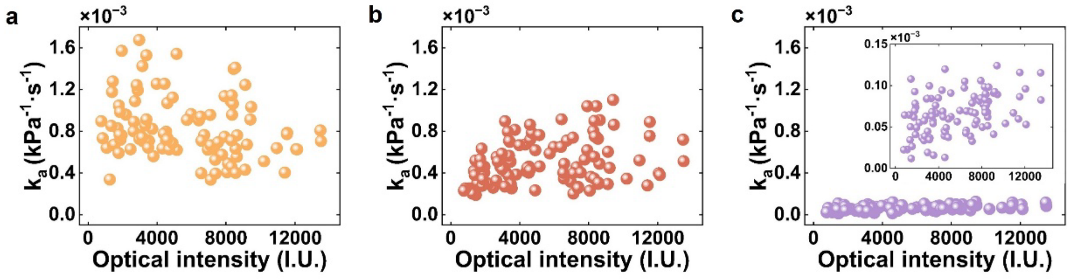

Supplementary Fig. 14. (a-c) Scatter diagrams of k_a versus I_0 of three olefins of single ZSM-5 nanoparticles.

15. The relationship between k_d, K_A and protonation energies

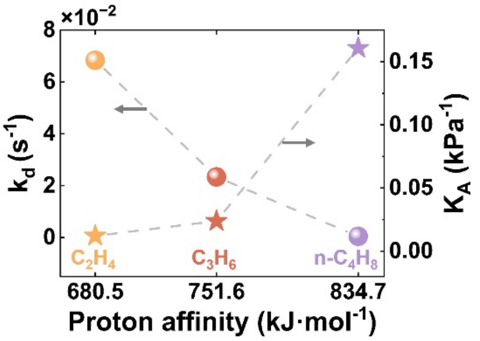

Supplementary Fig. 15. The experimentally determined k_d and K_A values of three olefins as a function of the their theoretically calculated protonation energies.

16. Determination of the adsorption and desorption activation energies of C_2H_4

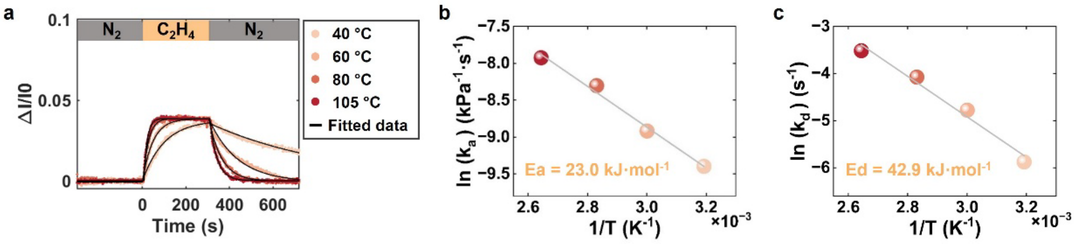

Supplementary Fig. 16. (a) The normalized optical intensity curves under different temperatures of C_2H_4. **(b-c)** Determination of the adsorption and desorption activation energies of a single ZSM-5 particle.

17. Determination of the adsorption and desorption activation energies of n-C_4H_8

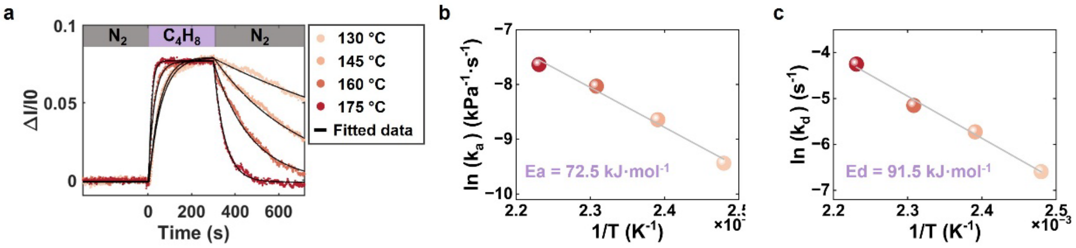

Supplementary Fig. 17. (a) The normalized optical intensity curves under different temperatures of n-C_4H_8. **(b-c)** Determination of the adsorption and desorption activation energies of a single ZSM-5 particle.

18. Relationship between temperatures and alkene adsorption amounts

To assess the effect of temperature on alkene adsorption amount, C_2H_4 adsorption experiments were conducted on ZSM-5 over a broader temperature. The results show that the equilibrium adsorption amount decreased by approximately 30 % for a single nanoparticle when the temperature was increased from 25 °C to 200 °C (Supplementary Fig. 18a and 18b), consistent with the statistical results from 156 particles (Supplementary Fig. 18c). So the temperatures selected (40 °C, 60 °C, 80 °C, 105 °C) were carefully chosen to optimize the accuracy of adsorption kinetic measurements.

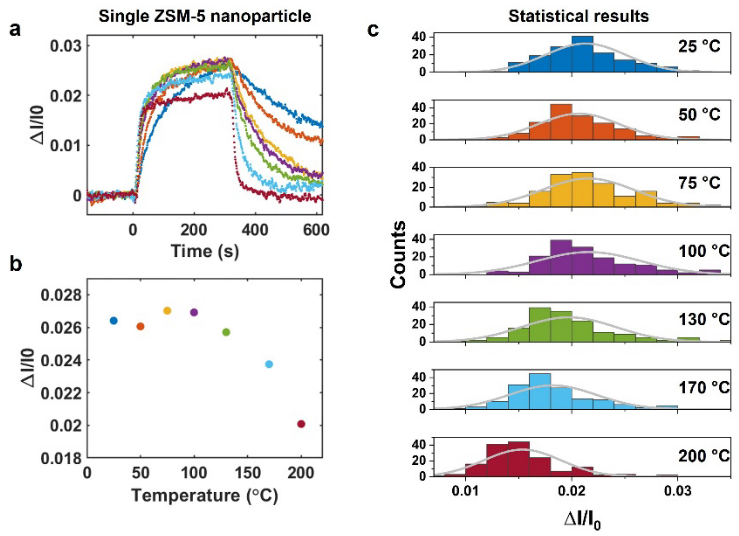

Supplementary Fig. 18. (a) The normalized optical intensity curves of C_2H_4 of single ZSM-5 nanoparticle from 25 °C to 200 °C during an adsorption-desorption cycle. **(b)** Plot of the equilibrium adsorption amount, extracted from panel (a), as a function of temperature. **(c)** The histograms of the equilibrium adsorption amount of 156 ZSM-5 particles under different temperatures.

19. Cyclic adsorption-desorption experiments of three olefin molecules

To evaluate the repeatability of the kinetic parameters, the temperature-series measurements were carried out on the same ZSM-5 nanoparticles in a single experimental sequence (Supplementary Fig. 19a). First, no appreciable changes were observed in the scattering intensity of the ZSM-5 nanoparticles before and after each measurement (Supplementary Fig. 19b), indicating that neither particle morphology nor optical properties were altered during the temperature-series experiments. Second, the adsorption behaviors of the three olefins at the highest temperatures used for Arrhenius analysis were highly reproducible across repeated measurements. As shown in Supplementary Fig. 19f both the extracted kinetic parameters and the equilibrium adsorption amounts were nearly identical when the adsorption–desorption sequence was repeated on the same single ZSM-5 nanoparticle.

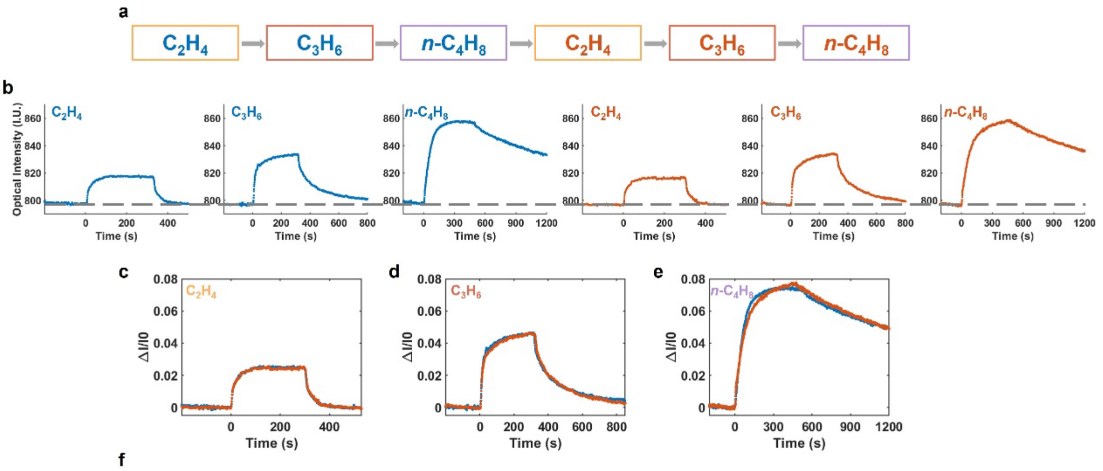

f

	k_{a1} ($kPa^{-1}\cdot s^{-1}$)	k_{a2} ($kPa^{-1}\cdot s^{-1}$)	k_{d1} (s^{-1})	k_{d2} (s^{-1})	K_{A1} (kPa^{-1})	K_{A2} (kPa^{-1})
C_2H_4	1.08E-4	1.08E-4	4.91E-2	4.98E-2	2.17E-3	2.20E-3
C_3H_6	2.95E-4	2.91E-4	7.30E-3	7.30E-3	4.04E-2	3.99E-2
n-C_4H_8	1.54E-4	1.52E-4	6.67E-4	6.38E-4	2.31E-1	2.38E-1

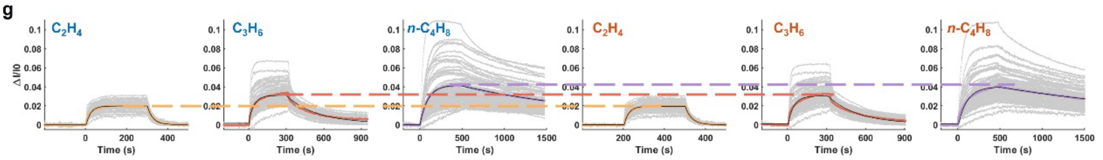

Supplementary Fig. 19. (a) Experimental flowchart for adsorption measurements of three olefins. **(b)** The original optical curves of a single ZSM-5 nanoparticle during the adsorption-desorption of three olefin molecules. **(c-e)** Representative single ZSM-5 particle adsorption-desorption kinetic curves for the cyclic adsorption process of three olefins. **(f)** The kinetic parameters extracted from (c-e). (**g)** The normalized optical intensity curves of the three olefin molecules on single ZSM-5 nanoparticles during an adsorption-desorption cycle. The gray lines represent the result obtained from a ZSM-5 nanoparticle, and the color lines represent the average response of all ZSM-5 nanoparticles.

20. Bulk characterizations of the HY and SSZ-13 particles

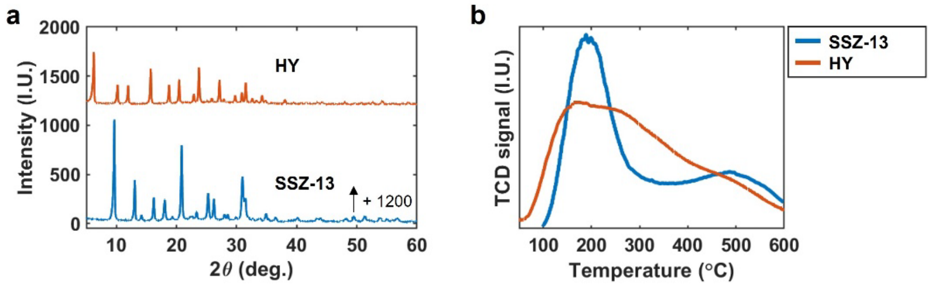

Supplementary Fig. 20. Characterization results of the HY and SSZ-13 particles used in this work. **(a)** XRD patterns. **(b)** NH_3-TPD spectra.

21. Comparison of the adsorption kinetics and thermodynamics of light alcohols

To study the generality of the conclusions, we investigated another important class of adsorbates—light alcohols (methanol, ethanol, and n-propanol)—whose molecular sizes are comparable to those of the light olefins but whose adsorption involves distinct hydrogen-bonding and acid–base interactions. Because these alcohols are liquid at room temperature, their vapor-phase adsorption on ZSM-5 was measured via nitrogen bubbling. As shown in Supplementary Fig. 21, these alcohols also exhibit the same confinement-dependent kinetic reversal, confirming that the observed behavior is not specific to olefins.

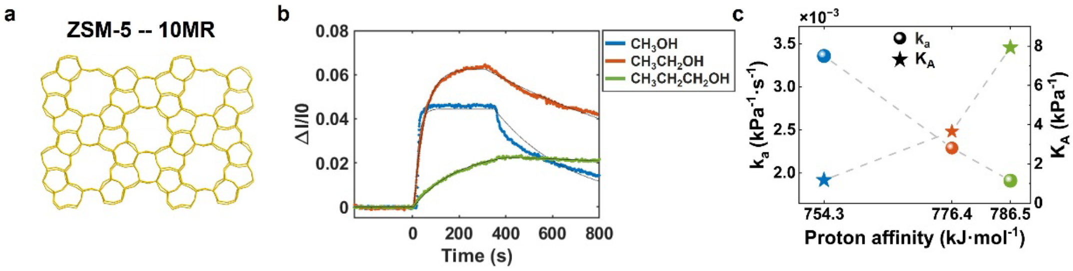

Supplementary Fig. 21. (a) MFI topology of ZSM-5 zeolite. **(b)** The normalized optical intensity curves of the three alcohol molecules on single ZSM-5 nanoparticle during an adsorption-desorption cycle at 100 °C. **(c)** The experimentally determined k_a (left axis) and K_A values (right axis) of three alcohol molecules as a function of their theoretically calculated protonation energies.

Supplementary Tables

1. Si/Al ratios of all ZSM-5 materials determined by ICP-OES

Prior to the measurement of adsorption kinetics, the zeolites with the same MFI framework, similar particle size range, but different Si/Al ratios were characterized. The ICP-OES confirmed the Si/Al ratios agreed well with the values provided by the supplier.

	W(Si)%	W(Al)%	$(Si/Al)_{ICP}$
ZSM-5-50	41.5125	0.7649	52.1
ZSM-5-400	43.2910	0.1008	412.6
Silicalite-1	44.6671	0.0167	2569.5

Supplementary Table 1. Si/Al ratios of all ZSM-5 materials used in this work, determined by ICP-OES.

2. The structural information of zeolites used in adsorption-desorption experiments

To investigate the adsorption behaviors of C_2H_4, C_3H_6, and n-C_4H_8 on zeolites with a range of pore sizes, including those both smaller and larger than the zeolite investigated in the original manuscript (Supplementary Table 2).

Sample	Framework Type	Featured number of rings (MR)	Pore size (nm)	CIRAK
SSZ-13	CHA	8 MR	0.38	Yes
ZSM-5	MFI	10 MR	0.51×0.55<br>0.53×0.56	Yes
HY	FAU	12MR	0.74	No

Supplementary Table 2. The structural information of the samples used in adsorption-desorption experiments. CIRAK: confinement-induced reversal of the adsorption kinetics.

References

1 Liu, S. S. *et al.* Optical Imaging of the Molecular Mobility of Single Polystyrene Nanospheres. *J. Am. Chem. Soc.* **144**, 1267-1273 (2022).

2 Frontmatter. In: *Absorption and Scattering of Light by Small Particles*) (1998).

3 Zhu, S. B. *et al.* Light-Scattering Detection below the Level of Single Fluorescent Molecules for High-Resolution Characterization of Functional Nanoparticles. *ACS Nano* **8**, 10998-11006 (2014).

4 Spoto, G. *et al.* IR STUDY OF ETHENE AND PROPENE OLIGOMERIZATION ON H-ZSM-5 - HYDROGEN-BONDED PRECURSOR FORMATION, INITIATION AND PROPAGATION MECHANISMS AND STRUCTURE OF THE ENTRAPPED OLIGOMERS. *J. Chem. Soc.-Faraday Trans.* **90**, 2827-2835 (1994).

5 Yamazaki, H., Yokoi, T., Tatsumi, T. & Kondo, J. N. Ethene oligomerization on H-ZSM-5 in relation to ethoxy species. *Catal. Sci. Technol.* **4**, 4193-4195 (2014).
